# Nickel-Driven Electrochemical Upgrading of Kraft Lignin to Value-Added Aliphatic and Phenolic Products

**DOI:** 10.3390/molecules30122544

**Published:** 2025-06-11

**Authors:** Yanbing Liu, Lucie M. Lindenbeck, Marcella Frauscher, Björn B. Beele, Bruno V. Manzolli Rodrigues, Adam Slabon

**Affiliations:** 1 Inorganic Chemistry, Faculty of Mathematics and Natural Sciences, University of Wuppertal, Gaussstraße 20, 42119 Wuppertal, Germany; yaliu@uni-wuppertal.de (Y.L.); lindenbeck@uni-wuppertal.de (L.M.L.); beele@uni-wuppertal.de (B.B.B.); 2 AC2T Research GmbH, Viktor Kaplan-Straße 2/c, 2700 Wiener Neustadt, Austria; marcella.frauscher@ac2t.at; 3 Wuppertal Center for Smart Materials & Systems, University of Wuppertal, 42119 Wuppertal, Germany

**Keywords:** lignin, depolymerization, electrocatalysis, nickel, *Green Chemistry*

## Abstract

The depolymerization of lignin represents a promising strategy for its efficient utilization as a precursor for industrial raw materials. However, achieving both high efficiency and environmental sustainability remains a significant challenge. In this study, we present an aqueous electrochemical approach employing nickel as an electrocatalyst, enabling both depolymerization and partial de-aromatization of Kraft lignin under mild reaction conditions. Using an aqueous sodium carbonate medium, room temperature and ambient pressure, we achieved lignin depolymerization over reaction times ranging from 5 to 20 h. Characterization by nuclear magnetic resonance (NMR) spectroscopy confirmed the formation of aliphatic products such as acetate and formate, while high-resolution mass spectrometry (HRMS) confirmed the formation of a wide range of phenolic compounds. The conversion of lignin into valuable aromatic and aliphatic compounds offers a promising pathway for the synthesis of a wide range of organic chemicals and their subsequent industrial utilization, thereby supporting the development of a more sustainable economy.

## 1. Introduction

Lignin, a complex macromolecule found in plants, constitutes approximately 15–30% of lignocellulosic biomass [1]. It consists of three main structural motifs: *p*-coumaryl alcohol, coniferyl alcohol, and sinapyl alcohol, which are linked via ether bonds [2,3] and is available as a by-product of the chemical pulping process and is conventionally discarded as waste, either discharged into black liquor or incinerated as a low-calorific-value fuel [4]. Besides works dedicated to the recovery of lignin and optimization of the machines’ energy consumption [5], many disposal practices not only result in significant resource wastage but also pose considerable environmental concerns, such as air pollution and wastewater high in biochemical oxygen demand [6]. As a polyphenolic macromolecule rich in aromatic structural units, lignin holds great potential as a precursor for the production of high-value chemicals and biofuels [1]. While lignin’s inherently robust and highly cross-linked spatial architecture provides mechanical strength and protective functions, it also leads to challenges for its (selective) depolymerization [7]. Nevertheless, recent advancements in catalyst utilization, such as copper [8], carbon [9] and silver [10], have shown promise in overcoming these limitations, offering new opportunities for lignin valorization. 

Conventional lignin depolymerization methods, such as pyrolysis [11] and hydrogenolysis [12], require additional energy input in the form of elevated temperatures and pressure. In contrast, electrochemical depolymerization of lignin under ambient conditions represents a promising approach for lignin valorization, facilitating the production of high-value-added chemicals while aligning with the principles of *Green Chemistry* [13]. A significant challenge in lignin depolymerization reactions is the condensation of phenolic intermediates into oligomers, a drawback that can be partially mitigated through the application of reductive pathways [14]. This method allows precise control over cell potential, enhances catalyst selectivity and activity, and enables reactions to proceed under mild conditions, including ambient temperature, atmospheric pressure, and environmentally benign solvents [8,9,10,15]. In accordance with *Green Chemistry* principles, electrochemical depolymerization of lignin using the biomass-derived solvent levulinic acid has been previously reported [8]. The depolymerization of lignin in levulinic acid, facilitated by a copper electrocatalyst at a potential of −1.7 V for 20 h, yielded monomeric and dimeric products predominantly composed of aryl ethers and phenolic groups [8]. These products have exhibited notable efficacy in corrosion resistance applications. Additionally, a method for the electrochemical depolymerization of Soda lignin in an aqueous sodium carbonate (Na₂CO₃) solution has been developed, employing carbon rods as working electrodes to facilitate the conversion the macromolecule into high-value-added aliphatic products [9]. Building upon this work, the electrochemical depolymerization of lignin using a silver-based electrocatalyst has also been explored, enabling depolymerization and partial de-aromatization of Soda lignin under environmentally benign conditions [10]. The primary products obtained included sodium levulinate, sodium acetate, and sodium formate as major aliphatic compounds, alongside various phenolic derivatives as aromatic products. 

Metal catalysts, particularly transition metals and their oxides, play a crucial role in catalytic processes due to their redox-active properties, which are essential for facilitating electron transfer [16]. These catalysts enable the cleavage of chemical bonds, leading to the formation of a diverse array of products. Nickel, in particular, is a well-established catalyst for hydrogenation reactions, owing to its partially filled *d* orbitals, which interact with the valence state of hydrogen to form bonding and anti-bonding states [17]. This characteristic enhances the activation of hydrogen sources, thereby improving catalytic efficiency [18]. Additionally, the moderate strength of hydrogen adsorption on nickel promotes its surface migration, facilitating the cleavage of C–O bonds while simultaneously inhibiting the recombination of reactive intermediates [18]. The electrochemical properties of nickel further contribute to its catalytic efficacy. Its standard reduction potential (Ni^2+^/Ni, −0.236 V vs. SHE) [19] is lower than that of noble metals such as platinum and palladium, indicating that nickel can drive catalytic reactions with higher energy efficiency. Furthermore, nickel is more abundant in the Earth’s crust compared to precious metals, making it a cost-effective alternative. It also exhibits excellent electrical conductivity [20], catalytic activity [21], and electrochemical stability [22]. Notably, nickel demonstrates strong corrosion resistance in both acidic and alkaline environments [23], can withstand a greater current density, and can shorten the reaction duration [24]. Nickel foam has the ability to cleave the benzylic C–O bond of the α-O-4 lignin model compound at room temperature and ambient pressure [25]. Nickel-coated carbon paper electrodes (Ni/CP) are able to reductively electrochemically break lignin model compound for the α-O-4 bond in the lignin, indicating that active Ni^0^ surface is crucial in facilitating proton transfer to enable the C–O cleavage [26]. The mechanism resembles Pd/C hydrogenation mechanism where the benzylic side approaches the catalytic surface [26]. These findings indicate that nickel plays a crucial role in the cleavage of the C–O bond in lignin model compounds. The reductive cleavage of the C–O bond primarily occurs due to the interaction between nickel ions and lignin model compound, as well as the in-situ generation of adsorbed hydrogen (H_ads_) on the electrode surface. 

**Figure 1:**
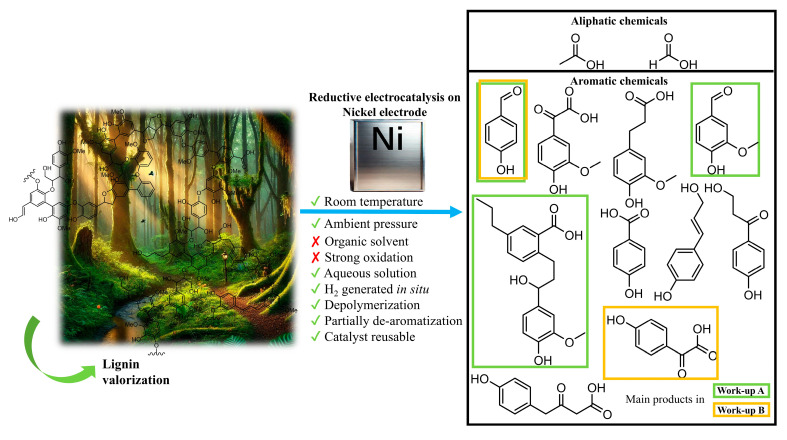
Illustration of the nickel-catalyzed electrocatalytic depolymerization and partial de-aromatization of Kraft lignin in an aqueous sodium carbonate solution. The process involves the application of a constant electrical current (−175 mA), driving the electrochemical reduction of lignin at room temperature and ambient pressure. The cleavage of lignin results in the formation of both aliphatic compounds, such as formate and acetate, and aromatic species. The figure emphasizes the role of nickel as an electrocatalyst in improving product distribution compared to previous systems and shows the distribution of products for different work-up methods.

In the Kraft process, a series of structural modifications like cleavage of the β-O-4 ether bonds, formation of C–C cross-linked structures, introduction of sulfur groups, and an overall reduction and condensation of structural elements occurs [27]. In this study, we have used nickel as an electrocatalyst for the reductive depolymerization of Kraft lignin, with a focus on its influence on product distribution (Figure 1). By exploiting the catalytic activity of nickel, the objective was to enhance the yield of high-value organic compounds from the depolymerization of lignin. We present a novel electrochemical approach for the depolymerization of Kraft lignin, conducted in an aqueous sodium carbonate solution using a nickel sheet as the working electrode under ambient temperature and atmospheric pressure.

## 2. Results

Electrochemical depolymerization was performed through chronopotentiometry at an electrical current of −175 mA, corresponding to a current density of −14.58 mA/cm^2^. After the depolymerization reaction, in both Work-up A and Work-up B, products were isolated through solid–liquid extraction, employing ethanol as the extractant. This choice was primarily driven by the low solubility of sodium carbonate in ethanol, thereby ensuring an efficient separation process. In addition, ethanol improves the overall efficiency and sustainability of the extraction [10], since its reusability allows for multiple extraction cycles with minimal loss of effectiveness, contributing to cost efficiency.

**Figure 2:**
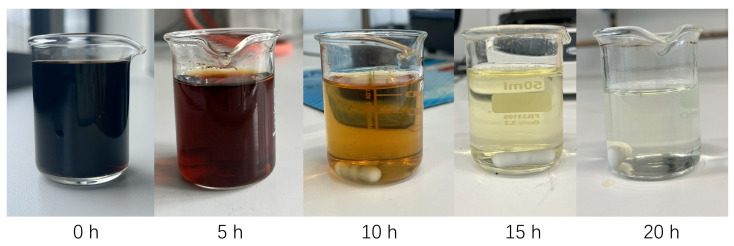
Electrochemical depolymerization of Kraft lignin in an aqueous sodium carbonate solution at −175 mA reacting for 0 h, 5 h, 10 h, 15 h, and 20 h, respectively. During depolymerization, the color of the solution changes over the reaction time and becomes clearer and more transparent as the reaction time increases. This observation can indirectly reveal the degree of depolymerization.

The dissolution of Kraft lignin in aqueous sodium carbonate solution results in the formation of a dark brown solution, as shown in Figure 2. It is well established that the reaction time and applied current have a significant impact on the decolorization of lignin solutions during depolymerization [9,10]. It is evident that with an increase in reaction time, the color of the Kraft lignin solution undergoes a transition from a dark brown hue to a light yellow one, becoming nearly colorless and transparent after 20 h. This observation not only points to the depolymerization process of Kraft lignin into low-molecular-weight substances but also provides an optical possibility to observe the reaction progress as a function of time.

The main reason for employing reductive depolymerization in this study was to mitigate peroxidation and undesirable repolymerization reactions that frequently occur during oxidative lignin depolymerization [8,28]. These side reactions often lead to the formation of high-molecular-weight by-products that are challenging to repurpose. By implementing reduction-based strategies, these issues were effectively minimized, resulting in the production of compounds predominantly enriched in aryl ethers and phenolic functional groups.

Table 1 shows the depolymerization yields for all reactions carried out in this investigation. Herein, yield has been defined as the proportion of ethanol-soluble depolymerized Kraft lignin product relative to the initial mass of Kraft lignin. While volatile products might have been generated during the course of the reaction, an increased quantity of products was found as the depolymerization process progressed with extended reaction times. Given that the reaction was conducted in an open system, the volatilization of these products could have contributed to the slight attenuation in the yield increase observed beyond 10 h of reaction time. In addition, a decrease in the volume of the reaction mixture was observed over the course of the reaction, which might be attributed to water splitting.

**Figure 3:**
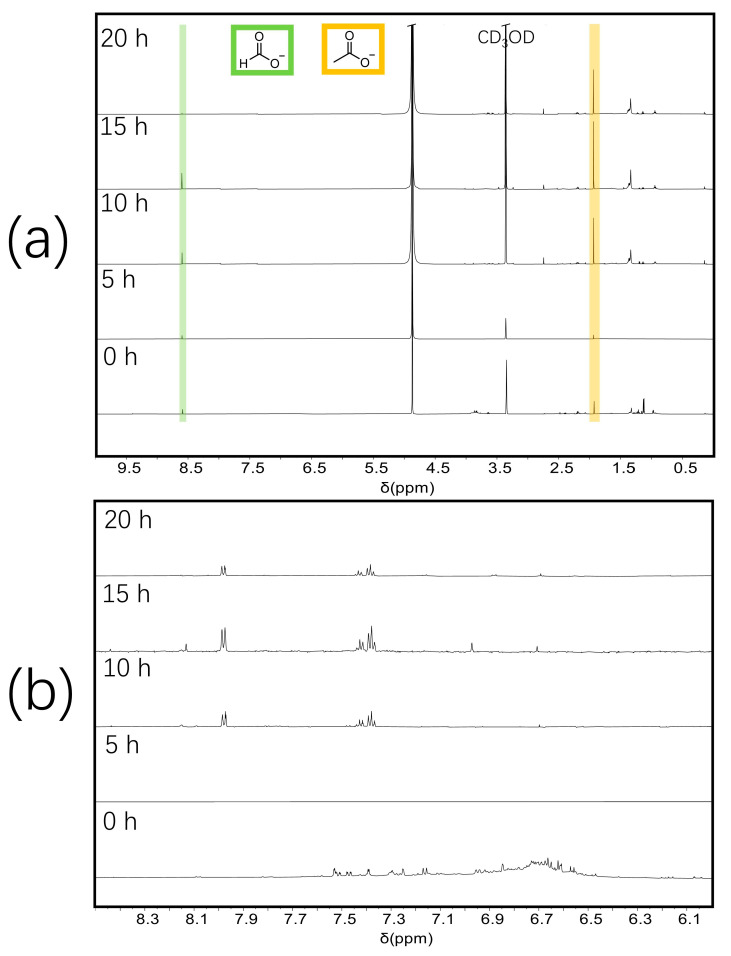
(**a**) ^1^H NMR spectra (CD_3_OD, 600.13 MHz) of depolymerized Kraft lignin for different reaction times: 0 h, 5 h, 10 h, 15 h, and 20 h obtained from Work-up A. Formate (green) and acetate (orange) were identified as aliphatic products [6,7]. (**b**) Enlarged section from 6.0 to 8.5 ppm of ^1^H NMR spectra (CD_3_OD, 600.13 MHz) of depolymerized Kraft lignin at different reaction times.

The usual limited yield in depolymerization processes, as observed in Work-up A, presents a significant challenge for the characterization of depolymerized lignin and poses obstacles to the subsequent scale-up for industrial production. To address this issue, Work-up B was designed in order to try to maximize the yield, resulting in substantially higher yields exceeding 40 wt%. In the ^1^H NMR spectra of Work-up A (Figure 3a), sodium acetate and sodium formate can be identified clearly. However, in Work-up A, the product was isolated as sodium salt, which exhibits limited solubility in ethanol. Consequently, the overall yield was relatively low. In contrast, acidification resulted in the protonated form of the product, which possesses greater solubility in ethanol, thereby significantly enhancing the yield compared to Work-up A. It should be noted, however, that the boiling points of acetic acid (118–119 °C) and formic acid (100.8 °C) are close to that of water. As a result, these volatile components are co-evaporated with water during reduced pressure distillation and thus are not detectable in the ^1^H NMR spectrum (Figure S1). Nevertheless, the distribution of the products in Work-up A is different from that in Work-up B, as shown in Table 2, so the products after electrochemical depolymerization of Kraft lignin can be obtained in different abundances by the two work-ups.

**Figure 4:**
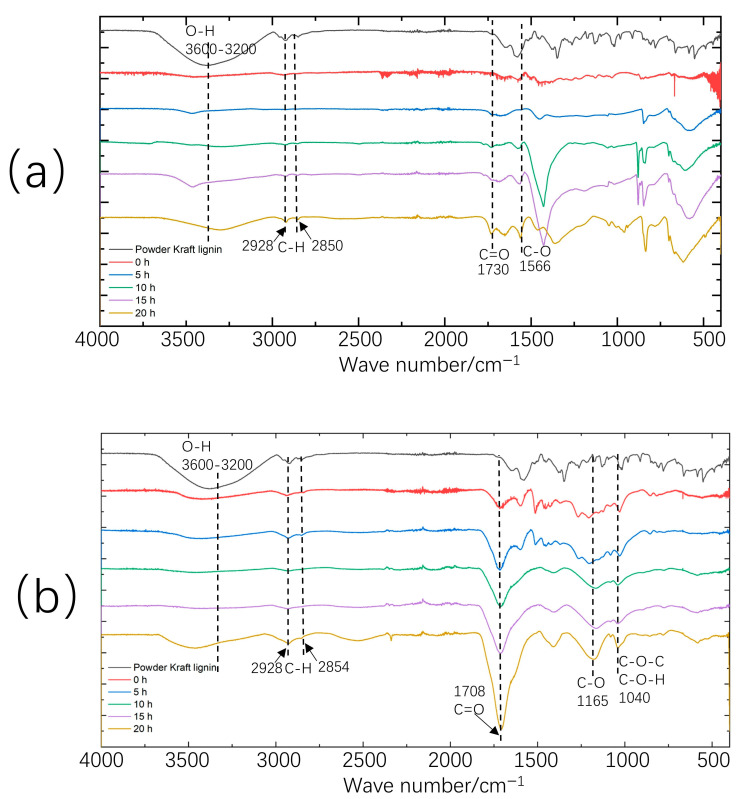
(**a)** FTIR spectra of Kraft lignin and depolymerized Kraft lignin from Work-up A at different depolymerization times. (**b**) FTIR spectra of Kraft lignin and depolymerized Kraft lignin obtained from Work-up B at different depolymerization times.

In Figure 4, the Fourier transform infrared (FTIR) spectra of the Ni-catalyzed depolymerized products from Kraft Lignin from Work-up A(a) and Work-up B(b) are shown according to different depolymerization times. All samples presented absorption bands typically found in lignin-based materials. Strong and broad absorption bands in the range of 3600–3200 cm^−1^ can be attributed to O–H stretching of the aromatic and aliphatic hydroxyl groups [29]. The bands centered at 2928 cm^−1^ are assigned to C–H stretching, while the bands centered at 2850 cm^−1^ can be related to the C–H stretching of methoxy groups (-OCH_3_) in aromatics [30]. In Figure 4a, the band at 1730 cm^−1^ can be attributed to the carbonyl (C=O) stretching vibration [9]. Bands at 1566 cm^−1^ are attributed to C–O stretching of carboxylic acid [30]. At 1420 cm^−1^, the C=C stretching of the aromatic skeleton and the C−H in-plane deformation of the aromatic ring can be observed. At 1458 cm^−1^, the C−H asymmetric deformation in the CH_2_ and CH_3_ groups, along with the C−H in-plane deformation, becomes clearly visible after 15 and 20 h of depolymerization (Figure 4a). In Figure 4b, the band at 1708 cm^−1^ is attributed to the carbonyl (C=O) stretching vibration. The bands at 1165 cm^−1^ indicate C–O stretching of the -COO- group. Furthermore, the bands at 1040 cm^−1^ indicate the presence of C–O–H and C–O–C groups [10]. 

Nuclear magnetic resonance (NMR) spectroscopy is a powerful analytical technique for identifying lignin structural groups [8,9,10]. To learn more about the presence of functional groups in Kraft lignin and depolymerization products, a ^1^H NMR study was performed as in Figure 3 and Figure S1 for Work-up A and Work-up B, respectively. In particular, two depolymerization products were identified: sodium formate and sodium acetate [9,10]. As shown in Figure 3, the peaks at 1.9 ppm were attributed to the proton in acetate, whereas the sharp peaks at 8.6 ppm were due to the proton in formate. The signals between 6.0 and 8.0 ppm can be attributed to aromatic protons [29]. Peaks for primary, secondary, and tertiary alkyl groups were observed around 0.8–1.7 ppm.

Due to the structural complexity of lignin, its depolymerization is essentially challenging, which can result in a broad distribution of products and reduced selectivity [31]. To gain a deeper understanding of the composition of the depolymerized lignin products, high-resolution mass spectrometry (HRMS) was employed for direct injection analysis, enabling the identification of the predominant aromatic compounds, as in Table 2. In Work-up A, 4-hydroxybenzaldehyde was the most dominant product, occupying the most product content. Notably, it was also the most abundant product in Work-up B. Other abundant compounds in Work-up A were 2-(3-hydroxy-3-(4-hydroxy-3-methoxyphenyl) propyl)-5-propylbenzoic acid and 4-hydroxy-3-methoxybenzaldehyde (vanillin). In contrast, the most abundant products in Work-up B were 2-(4-hydroxyphenyl)-2-oxoacetic acid. Other significant constituents included 4-(4-hydroxyphenyl)-3-oxobutanoic acid, 4-hydroxybenzoic acid, 2-(4-hydroxy-3-methoxyphenyl)-2-oxoacetic acid, and 3-(4-hydroxy-3-methoxyphenyl) propanoic acid. These results reaffirm the inherent heterogeneity of the depolymerization products. Our group has long advocated for the direct utilization of depolymerized lignin feedstock, rather than pursuing highly selective depolymerization toward single monomeric products. The use of such heterogeneous feedstocks is not only advantageous from a practical standpoint, by eliminating the need for extensive and often energy-intensive product separation, but also allows for scalable applications without the constraints of over-optimization. We have previously demonstrated the feasibility of this concept through the solvent-free synthesis of photoluminescent carbon nanoparticles from lignin-derived monomers [32]. Moreover, we recently proposed a “liquify-first” approach [33], which promotes the valorization of complex, unrefined depolymerized lignin-derived mixtures for industrial applications. 

**Figure 5:**
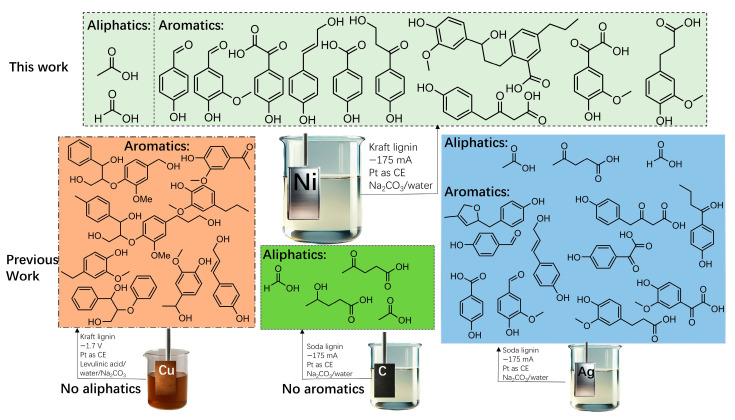
Comparison between the lignin depolymerization products obtained in this study and those reported in previous work involving the electrochemical reductive depolymerization of Soda and Kraft lignin under varying conditions [8,9,10]. The Figure illustrates the influence of different electrocatalysts (nickel, copper, carbon, and silver) and reaction parameters (current, reaction time, and electrolyte composition) on product distribution. The results underscore the critical role of electrocatalyst selection in determining the selectivity toward aliphatic or aromatic compounds.

Nickel demonstrates exceptional redox activity, which is essential for facilitating electron transfer processes in electrochemical reactions [34]. Its capacity for reversible oxidation and reduction enhances the cleavage of lignin’s C–O and C–C bonds, thereby promoting depolymerization [35]. The moderate hydrogen adsorption strength of nickel facilitates efficient hydrogen migration across the catalyst surface, thereby promoting the reduction of lignin intermediates while suppressing undesirable side reactions, such as repolymerization [36]. The metallic Ni site may have two key functions in the lignin depolymerization reaction: firstly, as an active site for hydrogenolysis of the C–O–C bond, and secondly as an active site for hydrogenolysis of the C–O–H bond on the side chain to the alkane chain [37]. This could explain why nickel contributes to the formation of aromatic products during electrochemical depolymerization of Kraft lignin, while not being as selective for aliphatic products as silver [10] and carbon [9]. To illustrate these results, Figure 5 presents a comparative analysis of lignin depolymerization products obtained in this study alongside data from previous electrochemical reductive depolymerization studies of Soda and Kraft lignin [8,9,10].

## 3. Materials and Methods

### 3.1. Materials

Kraft lignin (commercial name UPM BioPiva^TM^ 100) was obtained from UPM Biochemicals (Helsinki, Finland), with a reported molecular weight (M_w_) of 5000 g∙mol^−1^, ash content (700 °C) < 2%, and storage stability of 3–6 months, at 20 °C. Nickel sheet was obtained from Heraeus Feinchemikalien und Forschungsbedarf GmbH. Ag/AgCl (vs. saturated KCl); reference electrode and Pt mesh were purchased from Gaoss Union^®^. Sodium carbonate (99.5%, Grüssing GmbH, Filsum, Germany), hydrochloric acid (Thermo scientific, Darmstadt, Germany), ethanol (≥99%, Fisher Chemical, Loughborough, UK), and NMR SPECTRONORM^®^ Methanol D_4_ (99.8%, VWR Chemicals BDH^®^, Leuven, Belgium) were used as received. All aqueous solutions were prepared with ultrapure water, obtained from a Millipore system. 

### 3.2. Electrochemical Reactions

Electrochemical experiments were performed using an ATLAS 1131 Electrochemical Unit and Impedance Analyzer (Atlas Sollich, Kartuzy County, Poland). For a typical procedure, Kraft lignin (3 g∙L^−1^) was initially dissolved in 5 mL of 2 M sodium carbonate (Na_2_CO_3_) solution and stirred for 30 min. This was followed by the addition of 45 mL deionized water, with continuous stirring for 2 h until the solution became visibly clear, indicating complete dispersion. The depolymerization reaction was conducted in a three-electrode electrochemical setup. A nickel plate (2 cm width, 3 cm immersion depth, 12 cm^2^ working area) served as the working electrode. A saturated Ag/AgCl electrode was employed as the reference, while a platinum mesh was used as the counter electrode. Chronopotentiometry was carried out at a constant current of −175 mA for various durations (0 h, 5 h, 10 h, 15 h, and 20 h). All experiments were performed at room temperature under ambient pressure.

After completion of the electrochemical process, water was removed by evaporation under reduced pressure at 50 °C. The resulting residue was dried overnight at 105 °C, then finely ground and redissolved in ethanol in an amount equal to the original water volume. This mixture was stirred vigorously for 1 h, followed by filtration. Ethanol was then evaporated under reduced pressure at 50 °C, and the final solid product was dried at 60 °C for at least 24 h. This procedure was designated “Work-up A”.

“Work-up B” started from acidification of the solution to pH 2 when the electrochemical depolymerization was complete. The subsequent steps were consistent with those described in Work-up A. 

The yield of depolymerization was calculated as the following equation.

(1) $$Yield\;of\;products\;of\;depolymerized\;Kraft\;lignin=\frac{The\;weight\;of\;total\;products}{The\;weight\;of\;lignin}\times 100\%$$

### 3.3. Analytical Methods 

Nuclear Magnetic Resonance (NMR) spectroscopy was performed using both BRUKER Avance 400 MHz and Avance III 600 MHz instruments (Bruker Corporation, Billerica, MA, USA), with all measurements conducted at 300 K in deuterated methanol (CD_3_OD). Three types of probe heads were employed: a 5 mm broadband inverse probe with automatic frequency tuning, a 5 mm QNP probe, and a standard 5 mm broadband inverse probe. Tetramethylsilane (Me_4_Si) was used as the internal standard for chemical shift referencing. Unless otherwise noted, each ^1^H NMR spectrum was acquired over 16 scans with an inter-scan delay of 1.0 s.

Fourier transform infrared (FTIR) spectroscopy was carried out using a Nicolet iS5 spectrometer (Thermo Scientific, Waltham, MA, USA), equipped with an iD5 diamond attenuated total reflection (ATR) accessory. Spectra were recorded over the range of 4000–400 cm^−1^.

High-resolution mass spectrometry (HRMS) analyses were performed using a Direct Injection–Electrospray Ionization (DI–ESI) (Thermo Fisher Scientific, Bremen, Germany) technique to elucidate the structural features of the depolymerized Kraft lignin products. Samples (~200 μg/mL) were initially dissolved in 100 μL of water and diluted in methanol prior to analysis. Data acquisition was conducted on an Orbitrap-IQX mass spectrometer (Thermo Fisher Scientific, Bremen, Germany) equipped with an electrospray ionization (ESI) source. Measurements were performed in both positive (ESI⁺) and negative (ESI⁻) ion modes, with direct sample introduction at a flow rate of 5 µL min⁻¹. Typical operating parameters for ESI⁻ mode included a source voltage of 3.0 kV, sheath gas flow of eight arbitrary units, capillary temperature of 275 °C, capillary voltage of −50 V, and a tubescope voltage of −130 V. Fragmentation patterns and structural elucidation were primarily based on negative ion mode data, supported by complementary ESI⁺ analyses. Data were processed using Xcalibur software (version 2.0.7) and interpreted with Mass Frontier (version 8.0), both from Thermo Fisher Scientific.

## 4. Conclusions

In summary, this study presents a nickel-catalyzed electrochemical strategy for the depolymerization and partial de-aromatization of Kraft lignin under mild and environmentally benign conditions. Using an aqueous sodium carbonate solution, the process effectively converted Kraft lignin into a broad spectrum of value-added aliphatic and aromatic compounds. Structural analysis via NMR revealed the formation of acetate and formate as major aliphatic products, while DI–ESI–HRMS identified a diverse array of phenolic compounds, underscoring the chemical richness of the resulting product stream. Rather than isolating individual compounds, our approach embraces the complexity of the depolymerized mixture. We advocate for the direct utilization of heterogeneous lignin-derived feedstocks in various applications, thereby eliminating the need for extensive product separation and its associated energy and cost burdens. Following our recent perspective on the “liquify-first” approach, we propose that such feedstocks can be employed in diverse applications, including: sustainable lubricants and tribological additives—benefiting from their polar and aromatic functionalities; renewable fuel additives—where oxygenated aromatics and aliphatics may enhance combustion properties; and carbon dot precursors—as demonstrated in recent work by our group on the synthesis of photoluminescent nanomaterials from lignin-derived monomers. By reframing lignin depolymerization as a route to multifunctional feedstocks rather than discrete chemicals, this work contributes to the development of scalable, cost-effective, and application-driven biorefinery strategies. Future studies focusing on tailoring catalyst systems and work-up conditions could further optimize product profiles for specific downstream applications, thereby enhancing the overall utility of lignin as a renewable resource.

## Figures and Tables

**Table 1:** Yields of depolymerization products relative to the mass of products obtained after ethanol extraction in Work-up A and Work-up B relative to the initial mass of Kraft lignin. All reactions were carried out at room temperature and ambient pressure with a constant current of −175 mA. The yield from Kraft lignin corresponds to the ethanol-soluble fraction of untreated Kraft lignin (i.e., without electrochemical depolymerization). The yield at 0 h refers to the ethanol-soluble fraction of Kraft lignin dissolved in an aqueous sodium carbonate solution and left for 20 h without undergoing electrocatalysis.

**Reaction Time**	**Kraft Lignin**	**0 h**	**5 h**	**10 h**	**15 h**	**20 h**
Yield of Work-up A [wt%]	0.9	1.5	1.8	3.1	4.9	5.7
Yield of Work-up B [wt%]	5.9	6.6	13.7	20.1	33.2	42.3

**Table 2:** Main phenolic products of depolymerization identified by Direct Injection–Electrospray Ionization–High-Resolution Mass Spectrometry (DI–ESI–HRMS).

*m/z*	121.0295	137.0246	149.0610	151.0402
Chemical structure	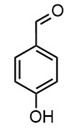	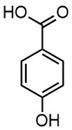	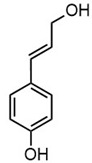	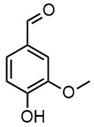
Name	4-hydroxybenzaldehyde	4-hydroxybenzoic acid	(*E*)-4-(3-hydroxyprop-1-en-1-yl)phenol; Cumarylalcohol	4-hydroxy-3-methoxybenzaldehyde; Vanillin
*m/z*	165.0195	165.0559	193.0507	195.0300
Chemical structure	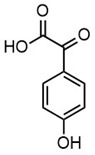	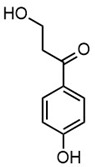	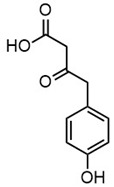	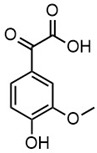
Name	2-(4-hydroxyphenyl)-2-oxoacetic acid	3-hydroxy-1-(4-hydroxyphenyl)-1-propanone	4-(4-hydroxyphenyl)-3-oxobutanoic acid	2-(4-hydroxy-3-methoxyphenyl)-2-oxoacetic acid
*m/z*	195.0664	343.1558		
Chemical structure	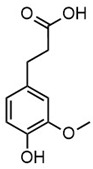	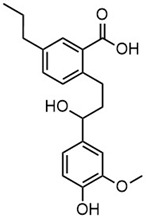		
Name	3-(4-hydroxy-3-methoxyphenyl)propanoic acid	2-(3-hydroxy-3-(4-hydroxy-3-methoxyphenyl)propyl)-5-propylbenzoic acid		

## Data Availability

The original contributions presented in the study are included in the article; further inquiries can be directed to the corresponding author.

## Supplementary Materials

The following supporting information can be downloaded at: https://www.mdpi.com/article/10.3390/molecules30122544/s1, Figure S1: ^1^H NMR spectra (400 MHz) of depolymerized Kraft lignin dissolved in CD_3_OD for different reaction times: 0 h, 5 h, 10 h, 15 h, and 20 h obtained from Work-up B.
