# Supplementary material for: Nickel-Driven Electrochemical Upgrading of Kraft Lignin to Value-Added Aliphatic and Phenolic Products
Source: Molecules. 2025 Jun 11;30(12):2544. doi: 10.3390/molecules30122544 (PMC12195935; doi:10.3390/molecules30122544)
Supplement: Supplementary file 1 [file molecules-30-02544-s001.zip › molecules-3672219-supplementary.pdf]

Supplementary Materials

Nickel-Driven Electrochemical Upgrading of Kraft Lignin to Value-Added Aliphatic and Phenolic Products

Yanbing Liu [1], Lucie M. Lindenbeck [1], Marcella Frauscher [2], Björn B. Beele [1], Bruno V. Manzolli Rodrigues [1,*] and Adam Slabon [1,3,*]

[1] Faculty of Mathematics and Natural Sciences, Chair of Inorganic Chemistry, University of Wuppertal, Gaussstraße 20, 42119 Wuppertal, Germany; yaliu@uni-wuppertal.de (Y.L.); beele@uni-wuppertal.de (B.B.B.); manzolli@uni-wuppertal.de (B.V.M.R.)

[2] AC2T Research GmbH, Viktor Kaplan-Straße 2/c, 2700 Wiener Neustadt, Austria; marcella.frauscher@ac2t.at

[3] Wuppertal Center for Smart Materials & Systems, University of Wuppertal, 42119 Wuppertal, Germany;

* Corresponding author e-mail: slabon@uni-wuppertal.de (A.S.); manzolli@uni-wuppertal.de (B.V.M.R.);

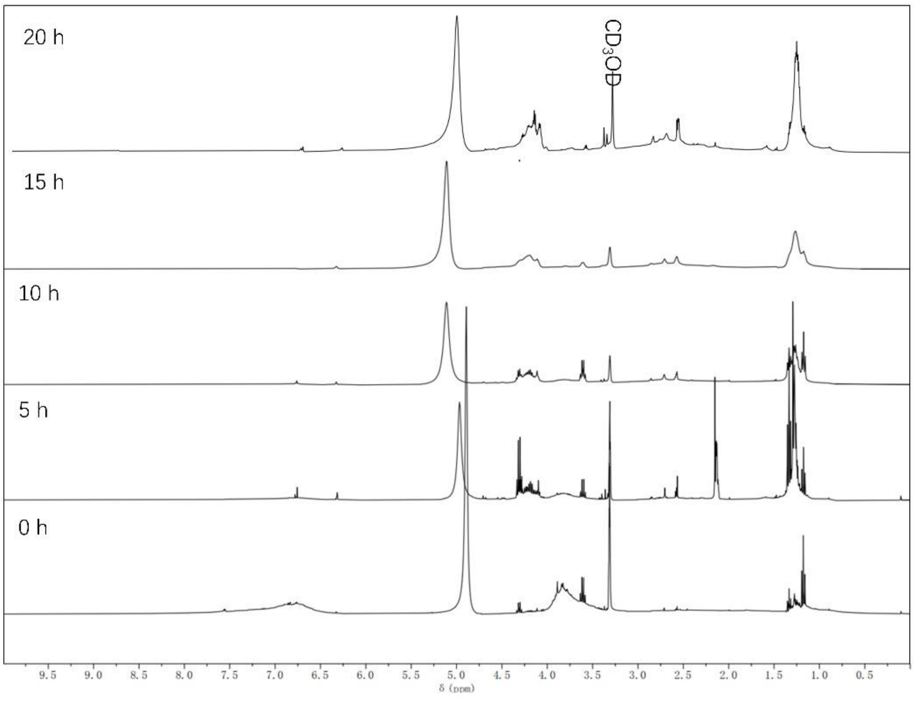

Figure S1. ^1H NMR spectra (600.13 MHz) of depolymerized Kraft lignin dissolved in CD_3OD for different reaction times: 0 h, 5 h, 10 h, 15 h, and 20 h obtained from Work-up B.

The signals between 6.0 and 8.0 ppm can be attributed to aromatic protons. Peaks for primary, secondary, and tertiary alkyl groups are observed around 0.8-1.70 ppm.
